# Biochemical basis of synergism between pathogenic fungus *Metarhizium anisopliae* and insecticide chlorantraniliprole in *Locusta migratoria* (Meyen)

**DOI:** 10.1038/srep28424

**Published:** 2016-06-22

**Authors:** Miao Jia, Guangchun Cao, Yibo Li, Xiongbing Tu, Guangjun Wang, Xiangqun Nong, Douglas W. Whitman, Zehua Zhang

**Affiliations:** 1 State Key Laboratory for Biology of Plant Diseases and Insect Pests, Institute of Plant Protection, Chinese Academy of Agricultural Sciences, Beijing, P. R. China; 2 Scientific Observation and Experimental Station of Pests, in Xilingol Rangeland, Ministry of Agriculture, xilin Gol League, Inner Mongolia, P. R. China; 3 College of Plant Protection, Shenyang Agricultural University, Shenyang, P. R. China; 4 School of Biological Sciences, Illinois State University, Normal, Illinois, USA

## Abstract

We challenged *Locusta migratoria* (Meyen) grasshoppers with simultaneous doses of both the insecticide chlorantraniliprole and the fungal pathogen, *Metarhizium anisopliae*. Our results showed synergistic and antagonistic effects on host mortality and enzyme activities. To elucidate the biochemical mechanisms that underlie detoxification and pathogen-immune responses in insects, we monitored the activities of 10 enzymes. After administration of insecticide and fungus, activities of glutathione-*S*-transferase (GST), general esterases (ESTs) and phenol oxidase (PO) decreased in the insect during the initial time period, whereas those of aryl acylamidase (AA) and chitinase (CHI) increased during the initial period and that of acetylcholinesterase (AChE) increased during a later time period. Activities of superoxide dismutase (SOD), catalase (CAT) and peroxidase (POD) decreased at a later time period post treatment. Interestingly, treatment with chlorantraniliprole and *M. anisopliae* relieved the convulsions that normally accompany *M. anisopliae* infection. We speculate that locust mortality increased as a result of synergism via a mechanism related to Ca^2+^ disruption in the host. Our study illuminates the biochemical mechanisms involved in insect immunity to xenobiotics and pathogens as well as the mechanisms by which these factors disrupt host homeostasis and induce death. We expect this knowledge to lead to more effective pest control.

Controlling insect pests, which continue to be agricultural, medical, and economic threats worldwide, requires constant human innovation^1^. One promising pest control method is to simultaneously attack insects with both an insecticide and an insect pathogen. This “dual-attack” approach can result in much higher insect mortality than using either of these methods independently^2^,^3^. Leveraging this combined approach to improve pest control requires a better understanding of the molecular and physiological mechanisms underlying the synergism between pesticides and pathogens. In this study, we attempted to understand the enzymatic consequences for the insect host of synergism between the insect-pathogenic fungus *Metarhizium anisopliae* and the insecticide chlorantraniliprole.

*Metarhizium anisopliae* (Deuteromycota: Hyphomycetes) is a well-known entomopathogen that has been successfully employed to control grasshoppers and other insect pests^4^,^5^,^6^. For example, applications of approximately 2.0 × 10^12^ to 1.0 × 10^13^ *M. anisopliae* conidia per hectare in nature can produce 80–98% mortality in locusts and grasshoppers in 7 to 21 d^4^,^5^,^7^,^8^,^9^. However, entomopathogenic fungi require long time periods to induce sufficient insect mortality. The dual-attack technique can overcome this problem, because the pathogen and insecticide often interact synergistically to both increase mortality and shorten the time until death in insects^2^,^3^,^10^,^11^,^12^. Chlorantraniliprole is a novel anthranilic diamide insecticide that causes feeding cessation, lethargy, muscle paralysis, and ultimately death^13^,^14^. Interestingly, both *M. anisopliae* and chlorantraniliprole harm insects, in part, by disrupting Ca^2+^ balance. Chlorantraniliprole activates the ryanodine receptor and affects calcium homeostasis by triggering Ca^2+^ release from the sarcoplasmic reticulum in host cells^15^. *M. anisopliae* produces numerous fungal secondary metabolites, including cyclic hexadepsipeptides, also known as destruxins, which induce membrane depolarization by opening Ca^2+^ channels, leading to tetanic paralysis and death of the host insect^16^,^17^. We hypothesized that a synergistic effect may exist between *M. anisopliae* and chlorantraniliprole and hence chose this specific pathogen and insecticide to study.

Insects defend against both insecticide and pathogen assault via multiple enzyme systems^18^,^19^,^20^,^21^. Multi-function oxidases (MFO), glutathione-*S*-transferase (GST) and general esterases (ESTs) are most commonly involved in defence processes in insects^22^. Because of their abundance, genetic diversity, broad substrate specificity and catalytic versatility, MFO, such as those involved in the P450 pathways in insects, are responsible for detoxification of natural and synthetic xenobiotics^23^. GST plays a pivotal role in detoxification and cellular antioxidant defences against oxidative stress by conjugating reduced glutathione to the electrophilic centres of natural and synthetic exogenous xenobiotics, including insecticides, allelochemicals and endogenously activated compounds^24^,^25^,^26^. ESTs perform important functions in insects through their involvement in the catabolism of the esters of higher fatty acids that influence flight as well in the degradation of inert metabolic esters^19^,^20^ and various xenobiotics, including insecticides. Changes in the activities of these enzymes are reflected in insect resistance to insecticides as well as in degradation of toxic molecules produced during *M. anisopliae* infection and, therefore, play a key role in the protection of insects against pathogens^18^,^21^.

Numerous other insect enzymes, including antioxidant enzymes such as superoxide dismutase (SOD), catalase (CAT) and peroxidase (POD)^27^ provide defences against pathogens and insecticides. Previous studies have demonstrated that these enzymes can be quickly up-regulated in response to xenobiotic threats and that increases in the activities of these enzymes are related to pesticide resistance and melanisation in insects^28^,^29^,^30^,^31^. The phenol oxidase (PO) cascade protects insects from microbial infections through its involvement in the melanisation of haemocytes attached to parasite surfaces^32^,^33^. Increased PO activity can strengthen an insect’s immunity to xenobiotics and promote wound healing^34^,^35^,^36^. Acetylcholinesterase (AChE) is a key enzyme catalysing the hydrolysis of the neurotransmitter acetylcholine in the nervous system of various organisms^37^. AChE is affected by organophosphate and carbamate insecticides, botanical insecticides and secondary fungal metabolites^38^,^39^,^40^,^41^,^42^. Conversely, aryl acylamidase (AA) has been found to confer resistance to chlorantraniliprole and fufenozide, whereas chitinase (CHI) confers resistance to fufenozide^43^,^44^. Although it is well known that both insecticide poisoning and fungal infections alter enzyme activities in insects^18^,^21^,^42^,^43^,^45^, few studies have explored the complex enzymatic consequences of simultaneous insecticide and pathogenic assaults in insects^46^, and no detailed investigations have been reported concerning the synergistic effect of *M. anisopliae* and insecticides on enzyme activities in insects.

The present study sought to understand the effects of simultaneous *M. anisopliae* infection and chlorantraniliprole poisoning on multiple insect enzyme systems. To this end, we evaluated the co-toxicity of chlorantraniliprole and *M. anisopliae* infection. Because many previous studies using the dual-attack strategy (simultaneous treatment with insecticide and pathogen) have used only one dose-level of each component^2^,^3^,^10^,^11^,^12^, we tested multiple dose-levels and measured the effects of different concentrations of chlorantraniliprole, *M. anisopliae* and their combination on MFO, GST, ESTs, PO, AChE, AA, CHI, CAT, POD and SOD activities in the locust *Locusta migratoria*. Our results establish a theoretical basis for the interaction between *M. anisopliae* and insecticides and should inform future studies investigating the mechanism by which *M. anisopliae* overcomes host immunity. We hope to use the information from this study to design better control methods for harmful insects.

## Results

### Virulence of chlorantraniliprole in combination with *M. anisopliae* against *L. migratoria*

The efficacy of chlorantraniliprole mixed with *M. anisopliae* against *L. migratoria* is presented in Table 1. The LC_50_ value of *M. anisopliae* alone against this insect was 0.15 mg/L (at ~7.5 × 10^6^ spores/mL). Mixing chlorantraniliprole with *M. anisopliae* in Treatments 1 and 2 resulted in higher mortality rates with LC_50_ values of 0.01 and 0.02 mg/L and co-toxicity coefficients of 1646 and 1619, respectively. In contrast, when formulated in Treatment 3, an antagonistic interaction was observed between chlorantraniliprole and *M. anisopliae*, resulting in a co-toxicity coefficient of 34.

In a separate experiment, *L. migratoria* was treated with *M. anisopliae* alone for 3 d before introducing wheat sprouts treated with chlorantraniliprole in Treatment 4, the resulting co-toxicity coefficient was 127 (Table 2).

### Effect of chlorantraniliprole and *M. anisopliae* on enzyme activities in *L. migratoria*

**Figure 1:**
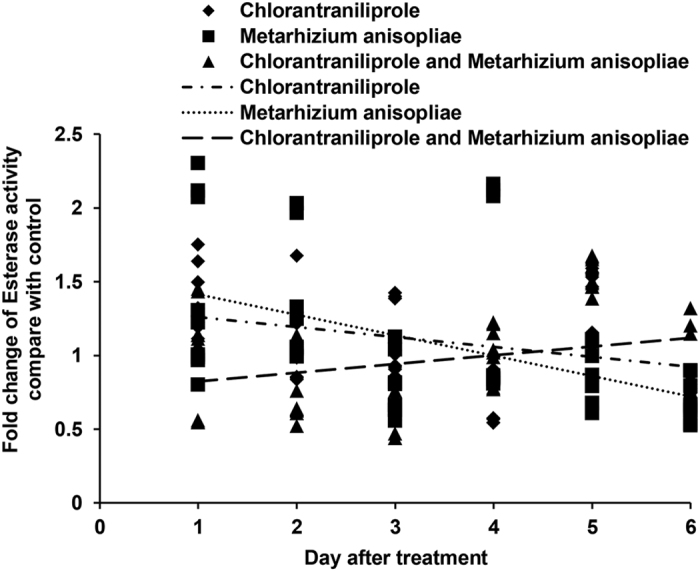
Linear-regression analysis of the interrelation between fold changes of esterases activities and different treatment days involving chlorantraniliprole, *Metarhizium anisopliae*, and *M. anisopliae* combined with chlorantraniliprole.

The activities of ESTs in *L. migratoria* are shown in Fig. 1. Chlorantraniliprole increased the activities of ESTs in *L. migratoria* during the initial post-treatment period; likewise, the activities of ESTs significantly increased during the initial period following *M. anisopliae* infection. However, the activities of ESTs markedly decreased after treatment with a mixture of *M. anisopliae* and chlorantraniliprole during the initial days of the experiment.

**Figure 2:**
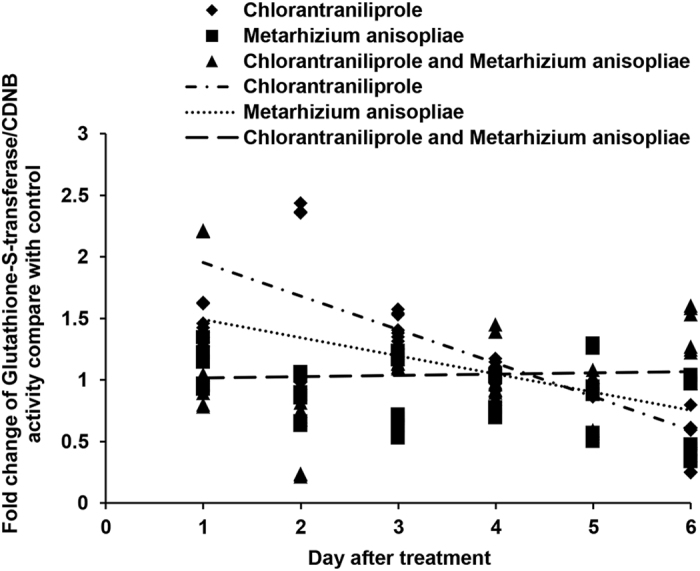
Linear-regression analysis of the interrelation between fold changes of glutathione-*S*-transferase/CDNB activity and different treatment days involving chlorantraniliprole, *Metarhizium anisopliae*, and *M. anisopliae* combined with chlorantraniliprole.

**Figure 3:**
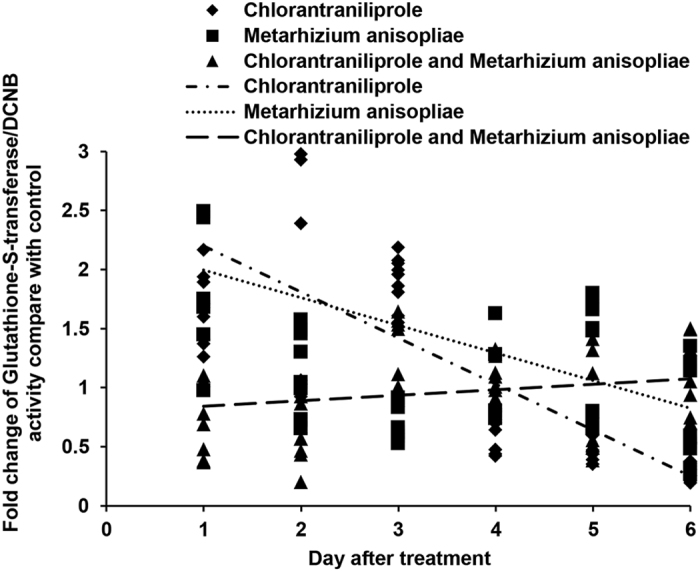
Linear-regression analysis of the interrelation between fold changes of glutathione-*S*-transferase/DCNB activity and different treatment days involving chlorantraniliprole, *Metarhizium anisopliae*, and *M. anisopliae* combined with chlorantraniliprole.

The activities of GSTs with different substrates were assessed, as shown in Figs 2 and 3. When treated with chlorantraniliprole, *L. migratoria* locust nymphs exhibited high GST activity (with CDNB or DCNB) during the early period, but this GST activity decreased during the later period. The activities of GSTs also increased during the initial period following *M. anisopliae* infection. However, GST activities decreased significantly during the initial period of the experiment when *L. migratoria* was treated with a combination of *M. anisopliae* and chlorantraniliprole simultaneously.

**Figure 4:**
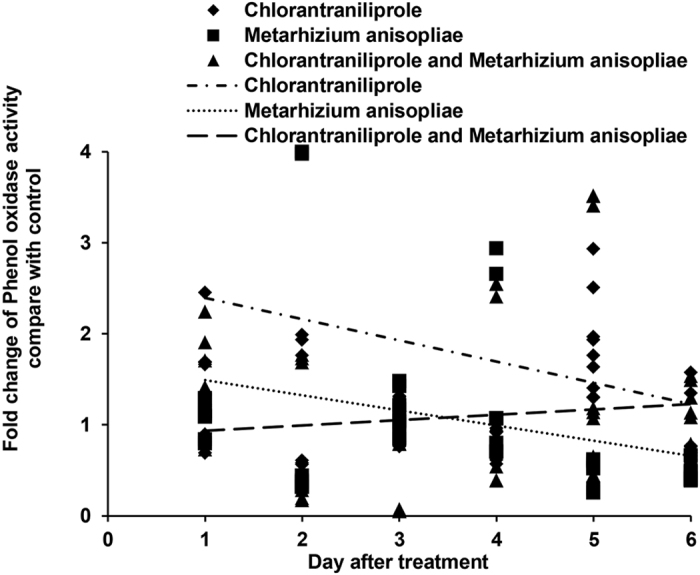
Linear-regression analysis of the interrelation between fold changes of phenol oxidase activity and different treatment days involving chlorantraniliprole, *Metarhizium anisopliae*, and *M. anisopliae* combined with chlorantraniliprole.

The activities of PO in *L. migratoria* after single and dual treatments were assessed, as shown in Fig. 4. When locust nymphs were treated with chlorantraniliprole alone, PO activities increased during the initial days of the experiment. When locust nymphs were treated with *M. anisopliae*, PO activities were high during the early period but decreased during the later period. In contrast, PO activities decreased during the initial days but increased during the later period when locust nymphs were treated with a combination of *M. anisopliae* and chlorantraniliprole.

**Figure 5:**
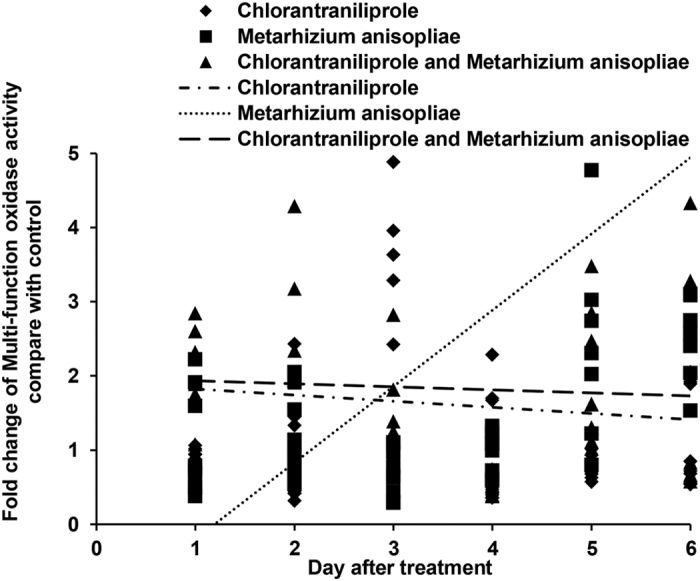
Linear-regression analysis of the interrelation between fold changes of multi-function oxidase activity and different treatment days involving chlorantraniliprole, *Metarhizium anisopliae*, and *M. anisopliae* combined with chlorantraniliprole.

The activities of MFO under single and dual treatment conditions were assessed, as shown in Fig. 5. MFO activity increased during the later period for locusts treated with *M. anisopliae* alone. MFO activity also increased during the later period when *M. anisopliae* and chlorantraniliprole were administered simultaneously; however, this increase in MFO activity was much lower than that obtained for treatment with *M. anisopliae* alone.

**Figure 6:**
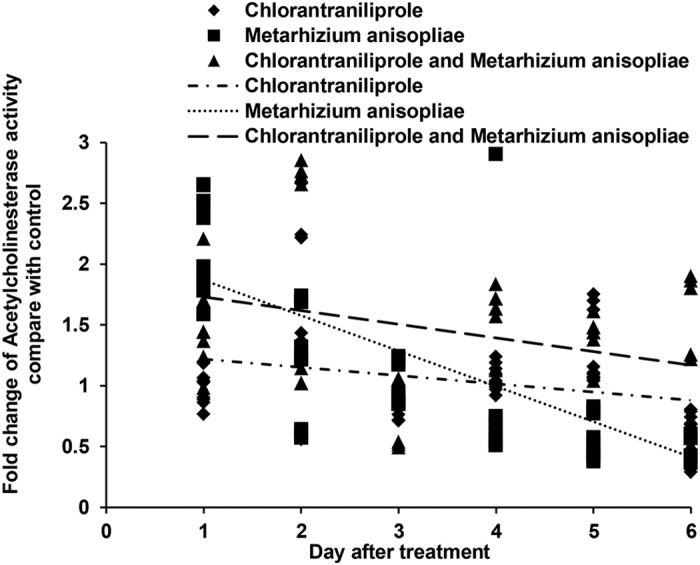
Linear-regression analysis of the interrelation between fold changes of acetylcholinesterase activity and and different treatment days involving chlorantraniliprole, *Metarhizium anisopliae*, and *M. anisopliae* combined with chlorantraniliprole.

The activities of AChE in *L. migratoria* under different treatment conditions are shown in Fig. 6. AChE activity increased in locusts during the early period following treatment with chlorantraniliprole alone. When treated with *M. anisopliae* alone, locust AChE activities increased during the early period but decreased during the later period. When locusts were treated with a combination of *M. anisopliae* and chlorantraniliprole, AChE activities increased throughout the entire period, except the third day.

**Figure 7:**
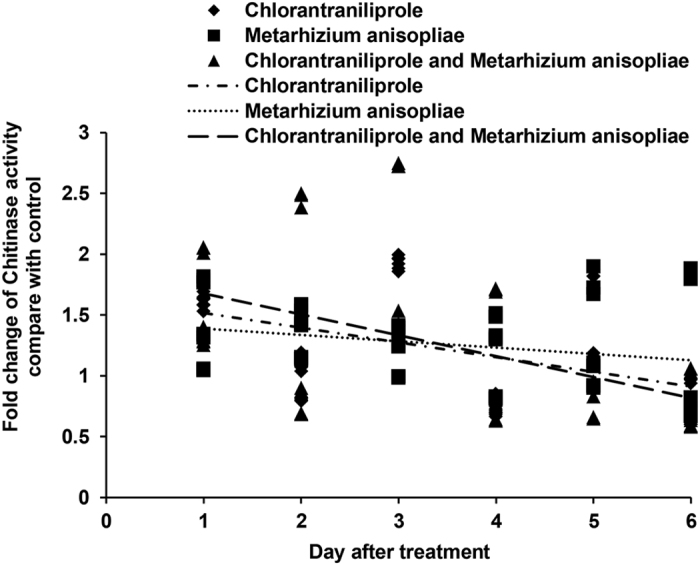
Linear-regression analysis of the interrelation between fold changes of chitinase activity and and different treatment days involving chlorantraniliprole, *Metarhizium anisopliae*, and *M. anisopliae* combined with chlorantraniliprole.

The activities of CHI (Fig. 7) in *L. migratoria* increased during the initial days of the experiment when locusts were treated with chlorantraniliprole alone. When locusts were treated with *M. anisopliae* alone, CHI activities increased throughout the entire analysis period. When locusts were treated with a combination of *M. anisopliae* and chlorantraniliprole, CHI activities increased during the early period but decreased during the later period.

**Figure 8:**
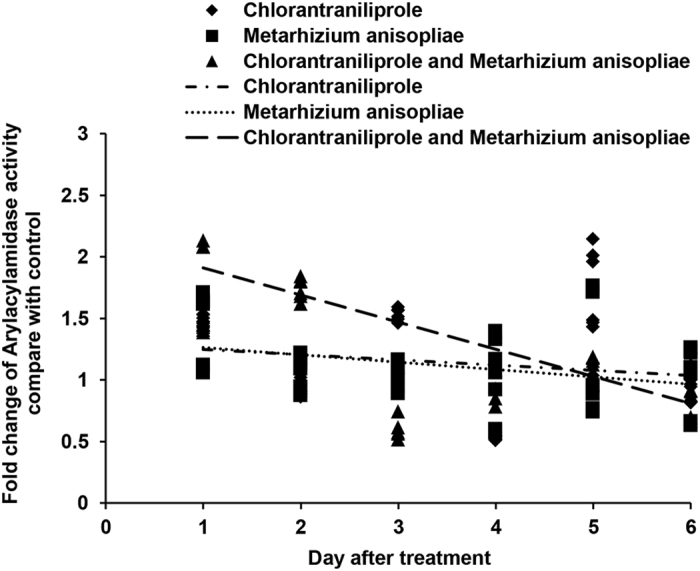
Linear-regression analysis of the interrelation between fold changes of aryl acylamidase activity and different treatment days involving chlorantraniliprole, *Metarhizium anisopliae*, and *M. anisopliae* combined with chlorantraniliprole.

The activities of AA (Fig. 8) increased during the initial days after independent treatment with chlorantraniliprole or *M. anisopliae*. AA activities also increased during the initial days after locusts were treated with a combination of *M. anisopliae* and chlorantraniliprole.

**Figure 9:**
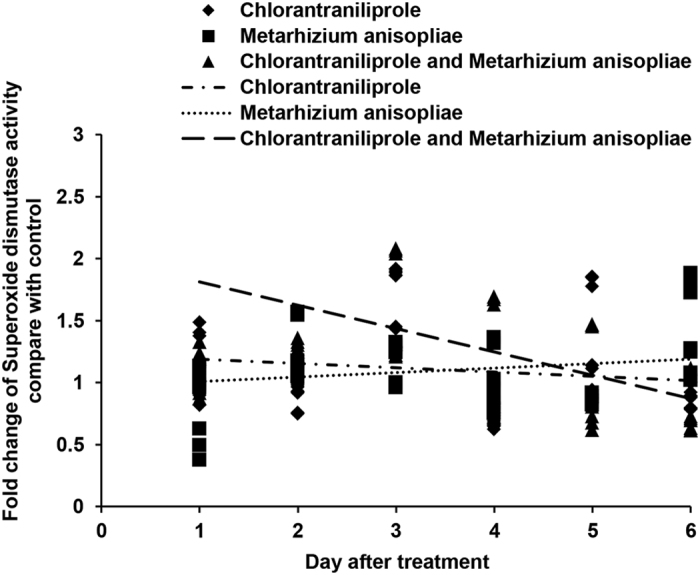
Linear-regression analysis of the interrelation between fold changes of superoxide dismutase activity and different treatment days involving chlorantraniliprole, *Metarhizium anisopliae*, and *M. anisopliae* combined with chlorantraniliprole.

**Figure 10:**
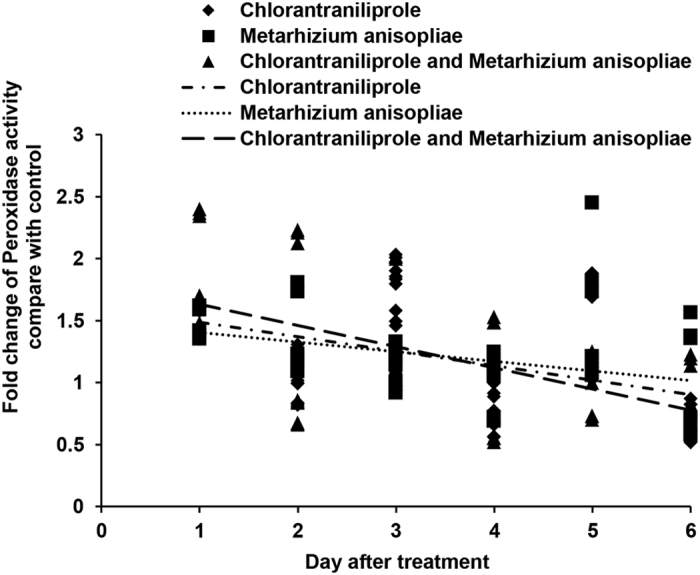
Linear-regression analysis of the interrelation between fold changes of peroxidase activity and different treatment days involving chlorantraniliprole, *Metarhizium anisopliae*, and *M. anisopliae* combined with chlorantraniliprole.

**Figure 11:**
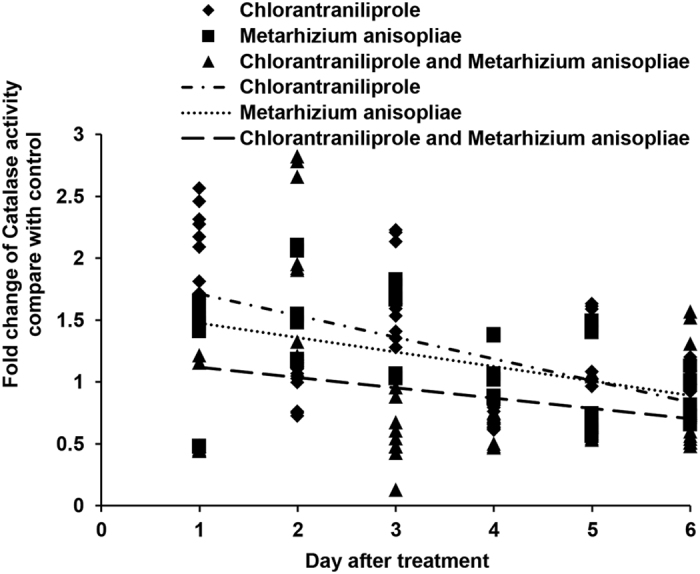
Linear-regression analysis of the interrelation between fold changes of catalase activity and different treatment days involving chlorantraniliprole, *Metarhizium anisopliae*, and *M. anisopliae* combined with chlorantraniliprole.

SOD, POD and CAT activities (Figs 9, 10, 11) increased during the initial days after independent chlorantraniliprole or *M. anisopliae* treatment. SOD and POD activities increased to high levels when locusts were treated with a combination of *M. anisopliae* and chlorantraniliprole; however, under these conditions, CAT activities decreased to low levels during the initial period, and, near the end of the experiment, CAT, SOD and POD activities decreased to low levels.

## Discussion

Previous studies have indicated that combinations of entomopathogenic fungi and insecticides can have synergistic, antagonistic or additive physiological and mortality effects on insects^10^,^12^,^47^. However, these investigations of fungal-insecticide interactions, mortality, and/or LC_50_ values were obtained using only one dose of pathogen or insecticide, such that the most efficacious synergistic formulations could not be determined^10^,^11^,^12^. The present study utilised co-toxicity coefficients to calculate insecticide incorporation rates and estimate the efficacies of different chlorantraniliprole+ *M. anisopliae* formulations. We found both synergistic and antagonistic interactions when chlorantraniliprole and *M. anisopliae* were administered together. Treatments 1, 2, and 4 revealed synergistic interactions with co-toxicity coefficients of 1646, 1619, and 127, respectively. These high co-toxicity coefficients, which were accompanied by insect mortalities >97% for some treatments, illustrate the effectiveness of this dual-attack method of insect pest control. In contrast, an antagonistic interaction (co-toxicity coefficient = 33) was observed for Treatment 3, possibly because of the high proportion of insecticide in this treatment. Our results demonstrate that chlorantraniliprole influences the virulence of *M. anisopliae* against *L. migratoria* relatively early in the initial infection period, producing a strong synergistic interaction with very high co-toxicity coefficients that vary depending on the proportions of the two agents and the time of application. As such, co-toxicity coefficients can be used to evaluate the effect of interactions between entomopathogenic fungi and insecticides, such that the best dose-combinations and time of application can be determined.

Our study also illuminated some of the biochemical consequences of a paired insecticide-pathogen challenge in insects. The accompanying enzymatic responses are numerous and complex, in part because they include attempts to detoxify the insecticide as well as the xenobiotic (the insect’s immune response to fungal attack) and because they involve biochemical/physiological disruptions due to the insecticide-and pathogen-induced pathology. Overall, most of the enzymes studied showed moderate to strong responses.

MFO, GST and ESTs are the major enzymes involved in detoxifying penetrating xenobiotics in insects^18^,^42^. Chlorantraniliprole has been found to increase the activities of ESTs and GST in Cry1Ac-susceptible and -resistant strains of *Helicoverpa armigera* caterpillars and to inhibit GST and MFO activities in *Plutella xylostella* larvae^43^,^48^. Sublethal doses of chlorantraniliprole have been observed to increase MFO and EST activities but to decrease GST activity in the Lepidoptera *H. armigera*, *Spodoptera exigua* and *Choristoneura rosaceana*^45^,^49^,^50^. In the present study, increased activities of GST and ESTs following chlorantraniliprole treatment suggest that these enzymes may act to detoxify this insecticide. Reported exceptions to these phenomena may be due to differences in the insect species as well as the concentrations of chlorantraniliprole used^45^,^48^,^50^.

Increased detoxifying enzyme activities against mycoses and other infections represent the insect’s response to bodily intoxication by metabolites or the host-tissue-degrading products of pathogens^51^. In the present study, we found that the activities of ESTs and GST were significantly increased during the initial period following *M. anisopliae* infection. MFO activity significantly increased during the later period following *M. anisopliae* infection. In Lepidopteran *Galleria mellonella* caterpillars, the activities of GST and ESTs in the haemolymph significantly increase during the first three days after *M. anisopliae* infection^18^. In the Hemipteran *Eurygaster integriceps*, the activities of GST and ESTs increase four to five days after infection with *B. bassiana* spores and secondary metabolites^42^. Alterations in the activities of GST and ESTs in *G. mellonella* and *L. migratoria* infected with entomopathogenic fungi are considered to constitute nonspecific body responses to integument damage, induction of additional isoenzymes or fungal toxins^21^,^51^,^52^. Based on these observations, GST and ESTs appear to participate in the defensive reaction of *L. migratoria* to *M. anisopliae* during the initial period of infection.

When *L. migratoria* were treated with a combination of *M. anisopliae* and chlorantraniliprole simultaneously, the activities of ESTs and GST decreased significantly during the initial period. However, MFO activity increased during the later period. Our results are consistent with those from a previously published investigation of the system components involved in insect detoxification. For example, when the Colorado Potato Beetle *Leptinotarsa decemlineata* is treated with a mixture of *M. anisopliae* and an organophosphate insecticide, EST and GST activities significantly decrease 2 d after inoculation^46^. Hall has reported that the pathogen sickens the pest, thereby lowering its chemical resistance, and the chemical, in turn, sufficiently weakens the pest and increases its susceptibility to pathogen infection^53^. Furlong and Groden reported that the interactions between fungi and insecticides are probably mediated during certain periods of the infection process, such as conidial attachment, conidial germination, cuticular penetration, or the initial proliferation of hyphal bodies in the haemocoel^54^. We believe that chlorantraniliprole can be used as a stressor in *L. migratoria* to increase its susceptibility to *M. anisopliae* and that toxic metabolites produced during the initial period of *M. anisopliae* infection may block the activation of detoxification components in the host.

PO is a key enzyme in insect immunity against pathogens. PO converts phenols to quinones, which subsequently polymerise to form melanin, which functions against both parasites and pathogens^32^,^34^. For example, in *Spodoptera exempta*, melanic larvae have higher PO activities and are more resistant to the entomopathogenic fungus, *Beauveria bassiana*, than non-melanic larvae^55^,^56^. Interestingly, high PO levels in insects might also increase resistance to insecticides. For example, insecticide-resistant diamondback moths display higher PO activities than susceptible moths^36^. In the present study, PO activities increased following treatment with either chlorantraniliprole or *M. anisopliae,* alone. In stark contrast, PO activities decreased significantly during the initial period after combined treatment with chlorantraniliprole and *M. anisopliae*, suggesting an antagonistic interaction of the insecticide and the pathogen in the host. Hiromori and Nishigaki have reported that mixed application of *M. anisopliae* and the insecticides teflubenzuron or fenitrothion inhibit PO activity in larvae of the beetle *Anomala cuprea*, and that granular cells are supressed^3^. These authors speculated that the observed interaction might be due to inhibition of the larval humoral defence and cellular immune systems. Moreover, joint treatment with *M. anisopliae* and an insecticide caused significant decrease in encapsulation intensity in the beetle *Leptinotarsa decemlineata*^46^. The interaction observed in our *L. migratoria* experiments might also be caused by PO inhibition and weakened humoral defence or cellular immune systems.

AChE is a key enzyme that terminates nerve impulses by catalysing the hydrolysis of the neurotransmitter acetylcholine in the nervous system of various organisms^57^. As such, AChE is the primary target site in the central nervous system for organophosphate and carbamate insecticides^41^. In our study, AChE activities decreased during the later period after *M. anisopliae* infection. A previous study has demonstrated the inhibitory effect of secondary metabolites on AChE in synapses of *B. bassiana*^42^. Inhibition of AChE causes acetylcholine to accumulate at synapses, such that post-synaptic membranes remain in a state of permanent stimulation; the result is paralysis, ataxia, a general lack of coordination in the neuromuscular system and eventual death^58^. In our experiment, however, AChE activities significantly increased throughout the entire experimental period, except for the third day, when locusts were treated with a combination of *M. anisopliae* and chlorantraniliprole. We speculated that the synergy between chlorantraniliprole and *M. anisopliae* affected AChE activity, thus mitigating symptoms of paralysis and ataxia.

CHIs are present in both fungi and insects. In fungi, they are involved in cell growth and division. In insects, they degrade chitin in the peritrophic membrane and exoskeletal cuticle and play an important role in moulting. Accumulating evidence suggests that CHI may also have roles in organismal defence against pathogenic fungi and parasites^59^,^60^,^61^. AA catalyses the hydrolysis of various anilide derivatives and esters and transfers an acetyl group to aniline, which acts as an acetyl acceptor^62^. In larvae of the diamondback moth, the specific activity of AA is significantly inhibited by chlorantraniliprole^43^. In our study, CHI and AA activities increased during the initial period after combined chlorantraniliprole and *M. anisopliae* treatment, thus helping the fungi to colonise the host. However, CHI activities decreased later in the experiment. We speculated that the synergy between chlorantraniliprole and *M. anisopliae* affected CHI and AA activities and disrupted the insect’s moulting process.

The major components of an insect’s antioxidant defence system include several antioxidant enzymes, such as superoxide dismutase (SOD), catalase (CAT) and peroxidase (POD)^27^. These enzymes remove damaging reactive oxygen species (ROS), such as superoxide anions (O_2_^•−^), hydrogen peroxide (H_2_O_2_), singlet oxygen molecules (O_2_^•^) and hydroxyl radicals (OH^•−^)^30^. ROS are regularly synthesised in the guts of insects following natural bacterial infections. The dynamic cycle of ROS generation and elimination appears to be a continuous and essential process in insects^63^,^64^. Studies in the grasshopper *Oxya chinensis* have shown increased SOD and CAT activities after treatment with phoxim, malathion and chlorpyrifos insecticides^65^,^66^. Likewise, SOD, CAT and POD activities have been shown to increase after selection by chlorpyrifos for eight generations in *Nilaparvata lugens* planthoppers^67^. SOD activity increases in the midgut of *Galleria mellonella L.* moth larvae on days 1–3 after infection by *Bacillus thuringiensis*, whereas CAT activity decreases during this time^68^. In our study, when *L. migratoria* were treated with *M. anisopliae* alone, CAT, SOD and POD activities increased during the initial period of infection. It should be noted that SOD and CAT, together, take part in stepwise oxygen reduction. Enhanced SOD activity in the infected nymph should elevate H_2_O_2_ concentrations and increase CAT activity. When chlorantraniliprole was applied in combination with *M. anisopliae*, SOD and POD activities increased but CAT activities decreased during the initial period post infection. We assumed that CAT was inhibited by the accumulation of superoxide radicals generated during the destruction processes. Research has shown that SOD1 protects calcineurin (CaN) from inactivation by ROS^69^. The enhanced activities of SOD and CAT then lead to the elimination of ROS^68^. In our study, the decreased activities of SOD, POD and CAT suggested a sharp decline in the ability to eliminate ROS during the later phase of infection. Large quantities of generated ROS can rapidly denature a wide range of biomolecules, including lipids, proteins and nucleic acids, thereby threatening virtually all cellular processes and leading to insect death^27^. Our results indicate a clear synergistic interaction between chlorantraniliprole and *M. anisopliae* infection when applied in combination.

Summarising locust enzyme responses to combined insecticide and pathogen treatment, it appears that chlorantraniliprole increased the probability of *M. anisopliae* infection and that *M. anisopliae* weakened *L. migratoria* defence against chlorantraniliprole. Our results suggest that combined treatment with chlorantraniliprole and *M. anisopliae* decreased GST, EST and PO activities during the initial post-infection period. Likewise, our results suggest that *L. migratoria* immunity to chlorantraniliprole and *M. anisopliae* was weakened by increased activities of AA and CHI during the initial post-infection period, whereas the activities of SOD, POD, CAT decreased during the later period. At the same time, combined treatment with chlorantraniliprole and *M. anisopliae* relieved the convulsions that normally accompany *M. anisopliae* infection by increasing the activity of AChE.

Based on previous and current results, we suggest that *L. migratoria* normally activates enzymatic defences against either pathogen or insecticide assault. Although these biochemical defences are sometimes successful, the combination of these two agents is able to overcome this defence. What is the chemical basis of this synergistic effect? One possibility is the disruption of Ca^2+^ concentrations in locust cells, given that both chlorantraniliprole and *M. anisopliae* are known to strongly disrupt Ca^2+^ balances in insects. For example, PO functions in an insect’s immune response to pathogens, but PO activity is regulated by Ca^2+^ concentration^70^,^71^. Hence, disrupting the Ca^2+^ balance could disrupt PO activity, thereby weakening the host’s immunity to *M. anisopliae*. Our results also reveal some of the complex biochemical processes that underlie the synergistic action of the two agents tested herein and illuminate the enzymatic mechanisms involved in insect immunity to pathogens and detoxification of insecticides. Understanding the response and activity of each enzyme alone and in combination will allow the design of novel and more effective means for pest control. Our results should provide guidance for future studies of the biochemical mechanisms underlying *M. anisopliae*’s disruption of host immunity.

## Methods

### Insects

Healthy 3rd-instar Oriental Migratory Locusts, *Locusta migratoria* (Meyen), were used in this study. Locusts were originally collected from Cangzhou, Hebei Province, China, then reared for 13 generations in the laboratory without exposure to insecticides. The eggs were hatched in a growth chamber maintained at 30 ± 2 °C and 60 ± 5% relative humidity (RH) for 2 wks. After hatching, the nymphs were fed fresh wheat sprouts and were maintained in a cage (60 × 50 × 70 cm) in the laboratory at 30 ± 2 °C and 60 ± 5% RH under a 14:10-h light:dark photoperiod.

### Fungus and synthetic insecticide

The entomopathogenic fungus *Metarhizium anisopliae* (Metschnikoff) Sorokin IMI330189 was cultured on potato dextrose agar yeast extract (PDAY) at 27 ± 1 °C and 75 ± 10% RH for 7–10 d under constant light. Conidia were harvested from culture plates by scraping the surface of the PDAY with a sterile mounted needle and were then placed into plastic centrifuge tubes containing 0.1% Tween in sterile water. An oscillator was used to break up any aggregates. The spore concentration was determined using an improved Neubauer haemocytometer and then adjusted to a concentration of 2.5 × 10^8^ spores/mL. For each experiment, this procedure was repeated to obtain a fresh suspension of spores. Chlorantraniliprole powder (96%) was provided by the Pesticide Science Group of the Institute of Plant Protection, Chinese Academy of Agricultural Sciences, Beijing, China. Preliminary trials showed that, at the doses tested (below) chlorantraniliprole did not significantly positively or negatively influence fungal growth on agar plates, nor did it influence fungal enzymes.

### Experimental procedures

#### Experiment I

Efficacy of chlorantraniliprole against *L. migratoria* under laboratory conditions. The leaf-dip bioassay method described by Shelton *et al*. and Liang *et al*.^72^,^73^ was adopted for the toxicity bioassay of chlorantraniliprole. Wheat sprout bundles (3-cm diameter) were cut near the roots and dipped in various concentrations of chlorantraniliprole (shown in Table 3) prepared with distilled water. A bundle dipped in distilled water was used as a control. Each bundle was dipped for 10 s and allowed to air dry at room temperature, and three replicates were performed for each concentration analysed. Each bundle was placed inside a separate plastic container (30 × 22 × 9 cm) in which 30 third-instar nymphs were confined. Nymph mortality was recorded at 24-h intervals for 6 d. Nymphs were recorded as dead if they did not move when probed with a camel-hair brush. *L. migratoria* were fed only wheat sprouts treated with chlorantraniliprole for 1 d; on the other 5 d they were fed fresh, non-contaminated wheat sprouts. We used a similar protocol to obtain insecticide-challenged locusts (chlorantraniliprole, 5 mg/L, 0.5 mg/L and 0.05 mg/L) for our enzyme studies. Any surviving nymphs were used for the enzyme activity analyses (see below).

#### Experiment II

Efficacy of *M. anisopliae* against *L. migratoria* under laboratory conditions. Five conidial suspensions were prepared at various concentrations in sterile water containing 0.1% Tween 80 (shown in Table 3). Sterile water containing 0.1% Tween 80 was used as a control. Third-instar nymphs were used for all experiments. A total of 30 third-instar nymphs were treated per conidial concentration, with 2 mL of each conidial suspension applied as a spray under a Potter Precision Spray Tower (Burkard Manufacturing, Rickmansworth, UK). Three replicates each of five concentrations were tested. After being subjected to the conidial suspension spray treatment, nymphs were confined in each container and fed fresh wheat sprouts. Mortality was recorded at 24-h intervals for 6 d; nymphs were recorded as dead if they did not move when probed with a camel-hair brush. We used a similar protocol to obtain *M. anisopliae*-challenged locusts (*M. anisopliae*, 2.5 × 10^8^, 2.5 × 10^7^ and 2.5 × 10^6^ spores/mL) for our enzyme studies. Any surviving nymphs were used for the enzyme activity analyses (see below).

#### Experiment III

Efficacy of chlorantraniliprole combined with *M. anisopliae* against *L. migratoria* under laboratory conditions. Chlorantraniliprole was combined with *M. anisopliae* in four dose-combinations for each of three proportions (Treatment groups 1, 2, & 3) (Table 3). A total of 30 third-instar nymphs were treated per solution by spraying under a Potter Precision Spray Tower with 2 mL of conidial suspension. Treatments were performed in triplicate. After being sprayed with a conidial suspension, nymphs were confined in containers and fed wheat sprouts treated with various concentrations of chlorantraniliprole for 1 d; on the other 5 d they were fed fresh, non-contaminated wheat sprouts. Mortality was recorded at 24-h intervals for 6 d; nymphs were recorded as dead if they did not move when probed with a camel-hair brush. We used a similar protocol to obtain chlorantraniliprole+ *M. anisopliae*-challenged locusts (1 mg/L + 2.0 × 10^8^ spores/mL, 0.1 mg/L + 2.0 × 10^7^ spores/mL and 0.01 mg/L + 2.0 × 10^6^ spores/mL) for our enzyme studies. Surviving nymphs were used for the enzyme activity analyses (see below).

#### Experiment IV

Efficacy against *L. migratoria* after 3-d exposure to *M. anisopliae* and chlorantraniliprole under laboratory conditions. Treatment 4: The concentrations of *M. anisopliae* solutions applied were similar to those used in Treatment 1 (shown in Table 3). After being sprayed with a conidial suspension, nymphs were confined in each container and fed fresh wheat sprouts for 3 d. On the 4th day, the nymphs were fed wheat sprouts treated with various concentrations of chlorantraniliprole (shown in Table 3, Treatment 4), and for the following 2 d the nymphs were fed fresh wheat. Mortality, which was assessed as described above for earlier experiments, was recorded at 24-h intervals for 6 d.

### Enzyme preparation

For enzyme assays, we took three 3rd-instar nymphs per insecticide x pathogen dose-combination with three replicates. Nymphs were homogenised in various solutions of 0.1 M ice-cold phosphate buffer (PBS) at different pH levels as follows: pH 7 (ESTs: 1.5 mL PBS with 0.1% TritonX-100; AChE and PO: 1.5 mL PBS), pH 7.3 (MFO: 1 mL PBS with 1 mM EDTA and 1 mM DTT; SOD, POD and CAT: 1.5 mL PBS), and pH 7.5 (AA, CHI and GST: 1.5 mL PBS). Extracted samples were centrifuged at 10,000 × *g* for 10 min at 4 °C. The resulting supernatant was transferred to a new Eppendorf tube and centrifuged at 15,000 × *g* for 20 min at 4 °C. The supernatant from this final centrifugation was used to determine enzyme activities and protein concentrations with five replicates were performed for each sample.

### Enzyme activity assays

The activities of ESTs were assayed using a modification of the method described by Han *et al*.^74^. Enzyme activity was determined by kinetic analysis using a microplate reader (Molecular Devices, LA, CA, USA), with 100 μL of 1-naphthyl acetate solution (10 mM), 100 μL Fast Blue RR salt (1 mM) and 90 μL PBS placed in each microplate well. The reaction was initiated by the addition of 10 μL of enzyme solution. Optical densities (ODs) at 450 nm were recorded at 25-s intervals for 10 min. All reactions were carried out at 27 °C. Enzyme activities were calculated as the rate of absorbance change per mg protein (mOD/min/mg).

GST activity was measured using a modification of the method described by Oppenoorth and Welling^75^. After pipetting 100 μL of 1-chloro-2,4-dinitrobenzene (CDNB) (20 mM) or 3,4-dichloronitrobenzene (DCNB) (40 mM) and 100 μL of GSH (40 mM) into microplate wells, we added 50 μL of enzyme solution (for DCNB) or 10 μL of enzyme solution and 90 μL of PBS (for CDNB). The OD values at 340 nm were recorded at 25-s intervals for 10 min. Enzyme activities were calculated as the rate of absorbance change per mg protein (mOD/min/mg).

MFO activity was assayed using a method modified from Hansen and Hodgson^76^. After pipetting 100 μL of p-nitroanisole (2 mM) and 50 μL of nicotinamide adenine dinucleotide 2′-phosphate reduced tetrasodium salt (9.6 mM) into microplate wells, we added 50 μL of enzyme solution. The OD values at 405 nm were recorded at 25-s intervals for 10 min. Enzyme activities were calculated as the rate of absorbance change per mg protein (mOD/min/mg).

AChE activity was assayed using a modification of the method described by Han *et al*.^74^. After pipetting 100 μL of 5,5′-dithiobis-(2-nitrobenzoic acid) (45 μM), 100 μL of acetylthiocholine iodide and 90 μL of PBS into microplate wells, we added 50 μL of enzyme solution. The OD values at 405 nm were recorded at 30-s intervals for 40 min. Enzyme activities were calculated as the rate of absorbance change per mg protein (mOD/min/mg).

PO activity was measured using a method modified from that described by Luo and Xue^77^. We pipetted 180 μL of catechol (10 mM) and 20 μL of enzyme solution into microplate wells and recorded the OD values at 420 nm at 1-min intervals for 1 h. Enzyme activities were calculated as the rate of absorbance change per mg protein (mOD/min/mg).

SOD, POD and CAT activities were determined using commercial assay kits (Nanjing Jiancheng, Nanjing, China) according to the manufacturer’s instructions. SOD, POD and CAT enzyme activities were measured in units of U/mg, U/mg and U/g, respectively.

CHI activity was measured by using a reducing-sugar assay. A 400-μL reaction mixture containing 300 μL of 1% (w/v) colloidal chitin (prepared based on the methods of Hsu and Lockwood^78^) and 100 μL of enzyme solution was incubated at 37 °C in an Eppendorf tube. After 4 h of incubation, the mixture was centrifuged at 8,000 × *g* for 5 min to precipitate the remaining chitin. We mixed 200 μL of supernatant with 80 μL of potassium tetraborate (0.8 M, pH 9.1). The reaction was terminated by boiling at 100 °C for 5 min, after which it was cooled to room temperature under running water. The mixture was mixed with 1.2 mL of dimethylamine borane (DMAB) (10 g DMAB diluted to 1,000 mL with distilled water and then diluted 10-fold with glacial acetic acid). This mixture was then incubated at 37 °C for 20 min and subsequently cooled to room temperature under running water. The absorbance was detected at 585 nm, with CHI activity measured as the change of absorbance per mg protein (ΔOD/min/mg).

A method modified from Tang *et al*.^44^ was used to assay AA activity. A reaction mixture comprising 50 μL enzyme solution, 50 μL p-nitroacetanilide (1.2 mM in absolute ethanol) and 150 μL PBS was incubated in water at 35 °C for 30 min. The reaction was terminated by boiling in water for 10 min, and the mixture was then centrifuged at 10,000 × *g* for 15 min. The p-nitroaniline released into the supernatant was measured at 405 nm, and boiled enzyme was used as a control. AA activity was expressed as the change of absorbance per mg protein (ΔOD/min/mg).

### Protein assay

Sample protein concentrations were estimated by using the method described by Bradford^79^. Bovine serum albumin was used for the calibration curve. Measurements were performed at 595 nm using a microplate reader with SoftMax Pro 6.1 software.

### Data Analysis

Co-toxicity coefficient values were calculated as described by Sun and Johnson^80^. Co-toxicity coefficients for the mixed formulation were calculated after calculating the LC_50_ of each incorporated component. Calculations were performed using the following equations: (1) toxicity index of agent = (LC_50_ of standard agent/LC_50_ of supplied agent) ×100; (2) theoretical toxicity index of the mixed formulation = (toxicity index of agent 1 × percentage of agent 1 in the mixed formulation) + (toxicity index of agent 2 × percentage of agent 2 in the mixed formulation); (3) co-toxicity coefficient = (actual toxicity index of the mixed formulation/theoretical toxicity index of the mixed formulation) ×100.

A co-toxicity coefficient of 100 indicates that the effect of the mixture is identical to that predicted from the proportions of the two components. A co-toxicity coefficient significantly greater than 100 corresponds to a synergistic effect. In contrast, when a mixture is characterised by a co-toxicity coefficient less than 100, the effect of the mixture is antagonistic.

LC_50_ values and concentration-mortality slopes for each bioassay were estimated via probit analysis^81^ using POLO-PC software^82^. Significant differences among LC_50_ values were determined on the basis of non-overlapping 95% confidence limits. In the figures, each symbol point represents average fold change values of five sub-replicates of enzyme activities compare with control. One-way ANOVAs were also used to analyse the activities of detoxification enzymes (ESTs, GST and MFO), protective enzymes (SOD, CAT and POD) and PO, AChE, CHI and AA in *L. migratoria* nymphs. Differences among means were compared by using the LSD test at *P* < 0.05.

## Additional Information

**How to cite this article**: Jia, M. *et al*. Biochemical basis of synergism between pathogenic fungus *Metarhizium anisopliae* and insecticide chlorantraniliprole in *Locusta migratoria* (Meyen). *Sci. Rep.* **6**, 28424; doi: 10.1038/srep28424 (2016).

## Figures and Tables

**Table 1:** The virulence of *Metarhizium anisopliae* fungi combined with chlorantraniliprole insecticide against *Locusta migratoria*.

**Treatment**		**Mortality (% ± SE)**	**LC-P Line (correlation ratio)**	**LC**_**50**_** ± SE (mg/L) (95% confidence limits)**	**CTC (co-toxicity coefficient)**	**Type of action**
Ch	20 mg/L	63.0 ± 1.9 a	y = 4.65 + 0.51x (0.94)	4.76 ± 2.59 (1.64–13.84)	—	—
	5 mg/L	55.6 ± 2.2 b				
	0.5 mg/L	31.1 ± 1.4 c				
	0.05 mg/L	31.9 ± 0.7 c				
	ck	6.7 ± 1.2 d				
Ma	2.5 × 10^8^ spores/mL	97.4 ± 1.3 a	y = 5.76 + 0.93x (0.94)	0.15 ± 0.04 (0.10–0.24)	—	—
	2.5 × 10^7^ spores/mL	68.8 ± 1.4 b				
	2.5 × 10^6^ spores/mL	15.6 ± 2.1 c				
	2.5 × 10^5^ spores/mL	13.0 ± 1.5 c				
	ck	6.5 ± 1.3 d				
T1	1 mg/L + 2.0 × 10^8^ spores/mL	97.5 ± 1.3 a	y = 6.15 + 0.59x (0.91)	0.01 ± 0.00 (0.01–0.02)	1646	synergistic
	0.1 mg/L + 2.0 × 10^7^ spores/mL	84.0 ± 2.7 b				
	0.01 mg/L + 2.0 × 10^6^ spores/mL	43.2 ± 2.4 c				
	0.001 mg/L + 2.0 × 10^5^ spores/mL	42.7 ± 1.8 c				
	ck	2.6 ± 1.3 d				
T2	2.5 mg/L + 1.25 × 10^8^ spores/mL	97.4 ± 1.3 a	y = 5.91 + 0.53x (0.85)	0.02 ± 0.01 (0.01–0.03)	1619	synergistic
	0.25 mg/L + 1.25 × 10^7^ spores/mL	61.0 ± 1.8 b				
	0.025 mg/L + 1.25 × 10^6^ spores/mL	48.1 ± 1.1 c				
	0.0025 mg/L + 1.25 × 10^5^ spores/mL	37.7 ± 1.2 d				
	ck	2.6 ± 1.3 e				
T3	4 mg/L + 0.5 × 10^8^ spores/mL	90.1 ± 1.3 a	y = 4.76 + 0.78x (0.85)	2.01 ± 1.04 (0.73–5.56)	33	antagonistic
	0.4 mg/L + 0.5 × 10^7^ spores/mL	17.1 ± 0.8 b				
	0.04 mg/L + 0.5 × 10^6^ spores/mL	8.5 ± 1.5 c				
	0.004 mg/L + 0.5 × 10^5^ spores/mL	8.9 ± 1.1 c				
	ck	2.6 ± 1.3 d				

Ch = chlorantraniliprole, Ma = *Metarhizium anisopliae*. “—” signifies “no co-toxicity.” T1, T2 and T3 = Treatment1, Treatment 2 and Treatment 3, respectively. Mortalities (% ± SE) contained within a column followed by the same letters are not significantly different (LSD test, *P* < 0.01).

**Table 2:** Mortality in *Locusta migratoria* following 3-d exposure to *Metarhizium anisopliae* followed by addition of chlorantraniliprole.

**Treatment**		**Mortality (% ± SE)**	**LC-P Line (correlation ratio)**	**LC**_**50**_** ± SE (mg/L) (95% confidence limits)**	**CTC (co-toxicity coefficient)**	**Type of action**
Ch	20 mg/L	63.3 ± 1.9 a	y = 4.73 + 0.37x (0.92)	5.24 ± 3.67 (1.33–20.68)	—	—
	5 mg/L	55.6 ± 5.9 a				
	0.5 mg/L	31.1 ± 5.9 b				
	0.05 mg/L	31.9 ± 4.4 b				
	ck	6.7 ± 1.3 c				
Ma	2.5 × 10^8^ spores/mL	87.5 ± 3.2 a	y = 5.38 + 0.80x (0.98)	0.33 ± 0.11 (0.18–0.62)	—	—
	2.5 × 10^7^ spores/mL	54.3 ± 4.6 b				
	2.5 × 10^6^ spores/mL	22.9 ± 1.1 c				
	2.5 × 10^5^ spores/mL	16.7 ± 1.7 c				
	ck	3.1 ± 0.1 d				
T4	1 mg/L + 2.0 × 10^8^ spores/mL	82.8 ± 2.8 a	y = 5.34 + 0.70x (0.99)	0.33 ± 0.14 (0.14–0.76)	126.54	synergistic
	0.1 mg/L + 2.0 × 10^7^spores/mL	55.6 ± 2.9 b				
	0.01 mg/L + 2.0 × 10^6^spores/mL	31.4 ± 5.2 c				
	0.001 mg/L + 2.0 × 10^5^ spores/mL	17.2 ± 5.2 cd				
	ck	3.1 ± 0.1 d				

Ch = chlorantraniliprole, Ma = *Metarhizium anisopliae*. “—”signifies “no co-toxicity.” T4 = Treatment 4. Mortalities (% ± SE) within a column followed by the same letters are not significantly different (LSD test, *P* < 0.01).

**Table 3:** Various concentrations of fungal pathogen and insecticide used in experiments (see Methods section).

**Experiment I**	**Experiment II**	**Experiment III**					
**Ch**	**Ma**	**Treatment 1 or Treatment 4 Ch:Ma = 2:8**		**Treatment 2 Ch:Ma = 5:5**		**Treatment 3 Ch:Ma = 8:2**	
20 mg/L	2.5 × 10^8^ spores/mL	1 mg/L	2.0 × 10^8^ spores/mL	2.5 mg/L	1.25 × 10^8^ spores/mL	4 mg/L	0.5 × 10^8^ spores/mL
5 mg/L	2.5 × 10^7^ spores/mL	0.1 mg/L	2.0 × 10^7^ spores/mL	0.25 mg/L	1.25 × 10^7^ spores/mL	0.4 mg/L	0.5 × 10^7^ spores/mL
0.5 mg/L	2.5 × 10^6^ spores/mL	0.01 mg/L	2.0 × 10^6^ spores/mL	0.025 mg/L	1.25 × 10^6^ spores/mL	0.04 mg/L	0.5 × 10^6^ spores/mL
0.05 mg/L	2.5 × 10^5^ spores/mL	0.001 mg/L	2.0 × 10^5^ spores/mL	0.0025 mg/L	1.25 × 10^5^ spores/mL	0.004 mg/L	0.5 × 10^5^ spores/mL
ck	ck	ck	ck	ck	ck	ck	ck

Ch = chlorantraniliprole, Ma = *Metarhizium anisopliae.*
